# Nutraceutical Supplementation Effects on Subjective Fatigue Symptoms in Myalgic Encephalomyelitis/Chronic Fatigue Syndrome: A Systematic Review

**DOI:** 10.7759/cureus.87178

**Published:** 2025-07-02

**Authors:** Emanuella M Brito, Leonardo Bonifanti, Rajvi Patel, Jailene Jimenez, Jacqueline Junco, Irina R Rozenfeld, Violetta Renesca, Amanpreet K Cheema

**Affiliations:** 1 Institute for Neuro-Immune Medicine, Nova Southeastern University Dr. Kiran C. Patel College of Osteopathic Medicine, Fort Lauderdale, USA; 2 Department of Biological Sciences, Nova Southeastern University Halmos College of Arts and Sciences, Fort Lauderdale, USA; 3 Institute for Neuro-Immune Medicine, Nova Southeastern University Dr. Kiran C. Patel College of Osteopathic Medicine, Davie, USA; 4 Department of Neurology, Nova Southeastern University Dr. Kiran C. Patel College of Osteopathic Medicine, Fort Lauderdale, USA; 5 Department of Nutrition and Neuro-Immune Research, Nova Southeastern University Dr. Kiran C. Patel College of Osteopathic Medicine, Fort Lauderdale, USA

**Keywords:** chronic fatigue syndrome, intervention, myalgic encephalomyelitis, nutraceuticals, supplements

## Abstract

Myalgic encephalomyelitis/chronic fatigue syndrome (ME/CFS) is a debilitating condition marked by severe, long-lasting fatigue and exhaustion that does not improve with rest. ME/CFS is reported in individuals of all ages and various racial, socioeconomic, and ethnic groups. This condition lacks standard treatment. Nutritional supplements and dietary interventions are often used to manage symptoms, but the efficacy of these interventions remains scarce in the current literature. This systematic review aims to evaluate and summarize recent evidence on nutrient supplementation and diet-based interventions in patients with ME/CFS sourced from clinical trial registries and article databases. Registries improve the quality, integrity, and transparency of clinical trials by providing a standardized platform for reporting study design and results and, thus, reducing the biases related to selective reporting practices. Systematic reviews using these registries, therefore, are an efficient pathway to acquire current medical evidence for use in clinical decision-making and the development of practice guidance in various fields. ClinicalTrials.gov, Medline, PubMed, Cochrane, and Web of Science were systematically searched for interventional studies in which patients suffering from ME/CFS supplemented or altered their diet. The results of this review showed several supplements that suggest improvement in patients’ symptomatology, including nicotinamide adenine dinucleotide (NADH), coenzyme Q10 (CoQ10), wasabi, and probiotics. However, many of these registered clinical trials did not employ the U.S. National Institutes of Health (NIH)’s National Institute of Neurological Disorders and Stroke (NINDS) suggested common data elements (CDEs). These standardized outcome-measuring tools allow the generalization and true comparison of the patient-reported outcomes.

## Introduction and background

Myalgic encephalomyelitis/chronic fatigue syndrome (ME/CFS) is a profoundly debilitating disease characterized by long-lasting fatigue and exhaustion that does not improve with rest. Currently, about 17-24 million people suffer from ME/CFS, with women having a two times greater risk of being affected than men [1].

The disease can be difficult to diagnose, as it often relies on the exclusion of other acute and chronic pathologies due to the condition’s varied symptomatology. Besides chronic fatigue, patients commonly experience sleep abnormalities, cardiovascular complications, endocrine imbalance, respiratory issues, cognitive dysfunction, pain, and post-exertional malaise (PEM), defined as a worsening of symptoms after physical and/or mental exertion [2,3]. Many ME/CFS patients are housebound or even bedridden for long periods of time, severely impacting their quality of life (QoL) [3]. The complex, multisymptom nature of ME/CFS, coupled with a lack of understanding of its pathophysiology, has hindered the development of clear treatments. The incidence of ME/CFS has been attributed to infectious illness, exposure to environmental toxins, viral infections, and stress [4]. However, no specific etiology has been determined [5]. Preclinical and human mechanistic studies have suggested dysfunctions in multiple organ systems influencing the myriad symptoms experienced by patients [4,5]. Biomarker, metabolomics, and genomic studies have highlighted abnormal functioning of various biochemical processes involving the metabolism of key nutrients [3,6-8].

Mitochondrial dysfunction is a recurring feature seen in ME/CFS that contributes to impaired energy production and symptoms of fatigue and PEM [9-11]. Nutraceuticals such as coenzyme Q10 (CoQ10) have been studied for their potential to improve mitochondrial dysfunction. In addition, oxidative and nitrosative stress pathways are implicated in the pathophysiology of ME/CFS, with increased levels of inflammatory cytokines exacerbating cellular damage. Several nutraceuticals have been studied to mitigate these inflammatory and oxidative stress effects [12]. Disruption of the gut mucosal barrier and subsequent bacterial translocation may further amplify immune and oxidative stress. Interventions such as probiotics and glutamine have been shown to restore gut integrity and reduce inflammation [9,13]. Moreover, integrative medicine approaches have demonstrated potential in ameliorating the debilitating physical and cognitive symptoms of ME/CFS [3,8,14-16]. Given the central role of mitochondrial and oxidative dysfunction in ME/CFS, nutraceuticals represent a promising therapeutic avenue. However, no systematic evaluation of nutrition/nutraceutical-focused registered studies in ME/CFS has yet been conducted.

This paper evaluates and discusses the most recent evidence regarding nutrient supplementation and diet-based interventions for patients with ME/CFS, using literature databases including Medline, PubMed, Cochrane, and Web of Science and, most importantly, clinical registries such as ClinicalTrials.gov. ClinicalTrials.gov, a registry of clinical trials run by the United States National Library of Medicine at the National Institutes of Health (NIH), provides information on the effectiveness of treatments while avoiding the information biases often linked with systematic reviews.

This article was previously presented as a meeting abstract at the 2023 Undergraduate Student Symposium (USS) at Nova Southeastern University Alvin Sherman Library, Research, and Information Technology Center.

## Review

Methods

This systematic review adhered to the Preferred Reporting Items for Systematic Reviews and Meta-Analyses (PRISMA) guidelines [17]. A systematic search was conducted to locate interventional studies with patients with ME/CFS. Studies that were included targeted nutrient supplementation and diet-based interventions.

The search process included the following steps: (i) A systematic search was conducted across ClinicalTrials.gov, Medline, PubMed, Cochrane, and Web of Science. The search was restricted to publication dates between 2010 and 2023. (ii) To identify relevant clinical trials pertaining to ME/CFS, ClinicalTrials.gov was searched with the “All Studies” status selected to include both completed and ongoing trials. The terms “ME/CFS”, “Chronic Fatigue Syndrome”, or “Myalgic Encephalomyelitis” for disease or condition were used. (iii) To limit the studies for interventions focused on nutritional supplementation or diet modification, the disease or condition was searched with the combination of the words “Nutrition”, “Nutraceutical”, and “Diet”. (iv) Medline, PubMed, Cochrane, and Web of Science were searched with advanced search builder showing (("benign myalgic encephalomyelitis" OR "chronic fatigue" OR "chronic fatigue and immune dysfunction syndrome" OR "encephalomyelitis, myalgic" OR "fatigue syndrome" OR "fatigue syndrome, chronic" OR "myalgic encephalomyelitis" OR "syndrome of chronic fatigue" OR "syndrome, chronic fatigue" OR "systemic exertion intolerance disease" OR "chronic fatigue syndrome") AND ("nutraceuticals" OR "nutriceutical" OR "nutriceuticals" OR "nutraceutical" OR "supplementation")). We screened full-text articles written in English and conducted on humans.

Eligibility Criteria

Studies were selected if they contained at least one nutraceutical or dietary intervention search term and at least one ME/CFS search term. They also had to satisfy the following inclusion criteria: (i) interventional studies published after 2010, (ii) study conducted on human participants aged 18 years or older, (iii) full text available in English, (iv) reporting of original research only, (v) diagnosis of ME/CFS follows Centers for Disease Control and Prevention (CDC) diagnosis criteria, and (vi) studies use nutraceuticals and/or diet intervention to target ME/CFS symptoms. Excluded studies included observational studies, narrative reviews, studies with multimodal interventions, and case reports, as well as studies not focused on ME/CFS as a primary diagnosis and studies including participants under 18 years of age or with comorbid conditions that confound ME/CFS symptomatology. Unpublished and ongoing clinical trials were also excluded.

Data Extraction

Relevant data were extracted from each of the studies. This includes the (i) study design, (ii) treatment intervention, (iii) treatment duration, (iv) country, (vi) number of participants, (vii) participants’ age, (viii) participants’ sex, (ix) adverse effects, (x) primary outcomes, and (xi) secondary outcomes.

Quality and Certainty Assessment

Quality and bias were assessed using the Delphi list for randomized controlled trials (RCTs). The Delphi list has been extensively evaluated and validated [18]. The Quality Assessment for Diverse Studies (QuADS) tool was used to assess open-label non-randomized studies. The QuADS tool is a modification of the QATSDD tool, which has shown substantial reliability for use in systematic review papers with multimethod health service research [19]. EMB, LB, and RP individually conducted the assessment, and no studies were excluded based on the results.

Results

**Figure 1:**
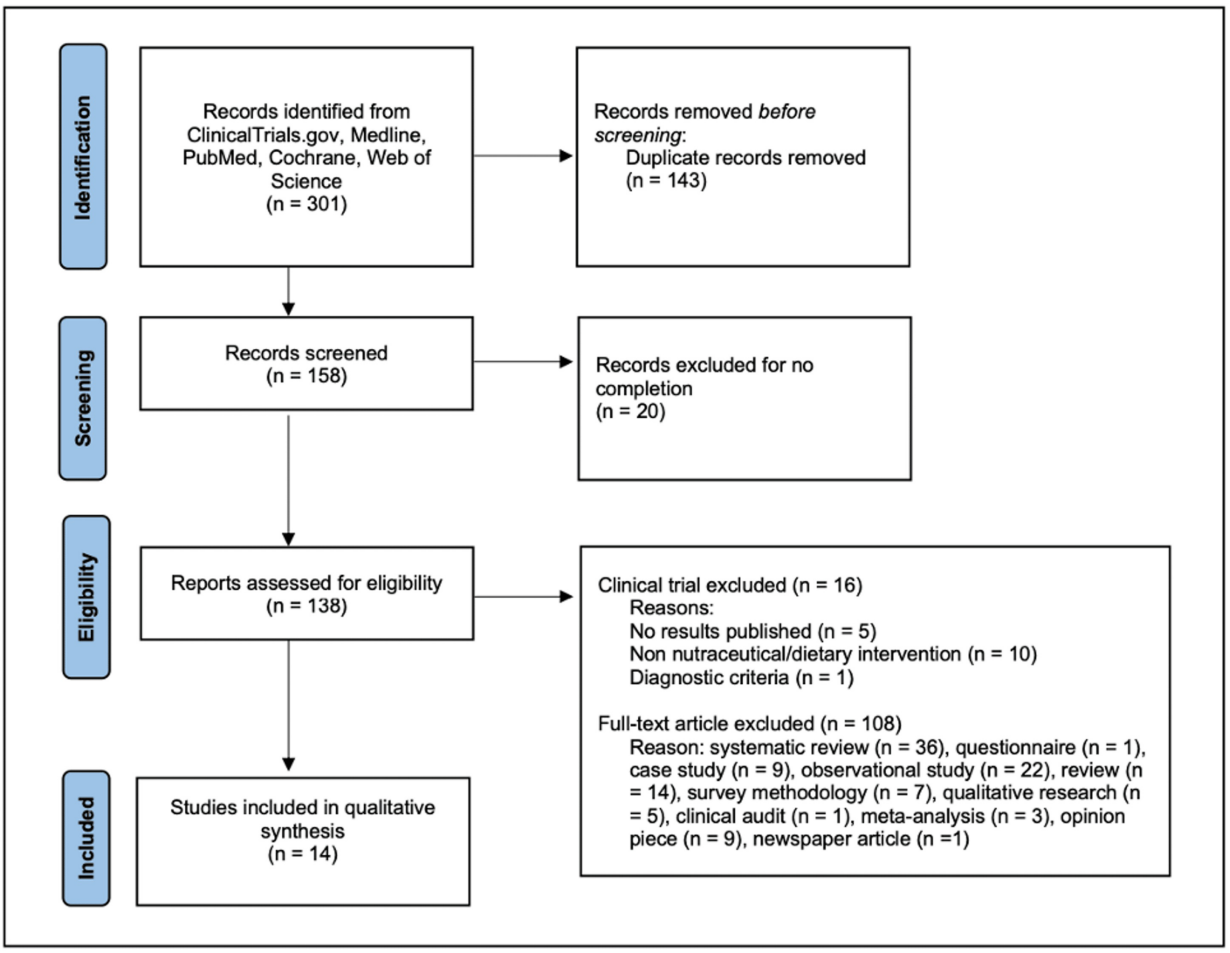
PRISMA flow diagram for study selection PRISMA criteria described in reference [17]. PRISMA: Preferred Reporting Items for Systematic Reviews and Meta-Analyses.

A total of 301 studies were retrieved from ClinicalTrials.gov, Medline, PubMed, Cochrane, and Web of Science. Duplicate studies were removed, yielding a total of 158 studies worldwide. Incomplete studies were removed, including three with unknown status, four withdrawn, eight recruiting, three actives but not recruiting, and two not recruiting yet, leaving 138 articles to be assessed for eligibility. Among the studies, 30 were completed clinical trials, of which five had no results published, 10 were not a supplemental or dietary intervention for ME/CFS, and one did not follow the ME/CFS diagnostic criteria by the CDC. Data from the 14 remaining studies were included in the systematic review. This selection process followed PRISMA guidelines summarized in Figure 1 [17]. Article screening was conducted independently by EMB and RJ, with a third reviewer, LB, breaking ties. Each individual reviewer reviewed all articles blindly.

Study Overview and Characteristics

The overview and characteristics of studies included in this review are presented in Table 1. A meta-analysis was not conducted due to substantial heterogeneity in study design, nutraceutical interventions, outcome measures, and follow-up durations. The included studies varied widely in the type, dosage, and duration of nutraceutical or dietary interventions, as well as in how outcomes were assessed, using different self-reported scales. All included studies were intervention-based. Following article selection, they were further classified based on study type. Seven were RCTs [20-26], three of which were proof-of-concept (POC) studies [21-23]. RCTs are designed to rigorously evaluate efficacy through random assignment to treatment arms, while POC studies are preliminary investigations assessing feasibility, biological activity, or early signals of efficacy. Among the RCTs, one followed a cross-over (CO) design [26], while the remaining five used a parallel design. In the CO design, participants received both the intervention and the control treatment in sequential periods, serving as their own controls and potentially reducing variability. In contrast, in a parallel design, each participant is assigned to only one treatment arm for the entire study duration. Seven of the studies were open-labelled pilot trials (OPTs) [27-33], two of which were also POC studies [30,31]. OPTs are exploratory studies without blinding, primarily assessing feasibility, safety, or preliminary outcomes. Fatigue was the primary outcome in 12 out of 14 studies [20,22-32]. Fatigue was measured using various self-reported questionnaires, including the Fatigue Impact Scale (FIS-40), the Chalder Fatigue Scale (CFQ-11), the Multidimensional Fatigue Inventory (MFI), a seven-point hedonic scale, the Visual Analog Scale (VAS), and the Fatigue Severity Scale (FSS) [20-33].

**Table 1:** Study overview and patient characteristics *Probiotics: (i) *Enterococcus faecium* and *Saccharomyces boulardii* (Enterelle); *Bifidobacterium longum*, *B. breve*, *B. bifidum*, and *B. infantis* (Bifiselle); (ii) *B. longum* AR81 (Rotanelle); (iii) *Lactobacillus casei* and *B. lactis* (Citogenex); and (iv) *L. rhamnosus* GG and *L. acidophilus* (Ramnoselle). CO: cross-over; CoQ10: coenzyme Q10; N/A: not applicable; NADH: nicotinamide adenine dinucleotide; NR: not recorded; OPT: open-labelled pilot trial; POC: proof-of-concept; RCT: randomized controlled trial; SD: standard deviation; Tx: treatment; DCA: dichloroacetate.

References	Year	Country	Study design	Treatment intervention	Treatment duration (weeks)	Reported side effects	Final number of participants		Age (years) mean (SD)		Sex, female %	
							Control	Tx	Control	Tx	Control	Tx
Barletta et al. [20]	2023	Italy	RCT	CoQ10 + alpha-lipoic acid	8	No reported adverse effects	58	116	50.3	51.7	51.1	48.9
Cash and Kaufman [30]	2022	USA	OPT, POC	Anhydrous enol-oxaloacetate (AEO)	6	Dyspepsia 2/23 500 mg BID, 2/24 1,000 mg BID and insomnia 1/26 500 mg BID	N/A	76	N/A	47	N/A	77.6
Castro-Marrero et al. [21]	2016	Spain	RCT, POC	CoQ10 + NADH	8	No reported adverse effects	34	39	49.1 (8.4)	49.3 (7.1)	100	100
Castro-Marrero et al. [22]	2021	Spain	RCT, POC	CoQ10 + NADH	12	Epigastralgia, dizziness	72	72	46.8 (6.5)	45.4 (7.8)	100	100
Castro-Marrero et al. [23]	2021	Spain	RCT, POC	Melatonin + zinc	16	No reported adverse effects	24	26	53.7 (9.6)	51.0 (10.2)	100	100
Castro-Marrero et al. [27]	2022	Spain	OPT	CoQ10 + selenium	8	No reported adverse effects	N/A	27	N/A	47.3 (1.5)	N/A	100
Comhaire [31]	2018	Belgium	OPT, POC	Sodium DCA	4	Generalized moderate tremor in one pt, which disappeared immediately after decreased DCA dose	N/A	22	N/A	43.3	N/A	63.3
Friedberg and Choi [24]	2022	USA	RCT	Hydrogen water	4	Headache, heartburn, loose stools, pain, and brain fog	11	11	53.6 (7.3)	40.3 (10.7)	81.8	75
Kan et al. [25]	2021	China	RCT	*Ginkgo* + *Cistanche*	8	No reported adverse effects	58	117 (59 high dose; 58 low dose)	50.7 (7.6)	50.5 (7.0) high dose; 51.5 (7.5) low dose	50	52.5% high dose, 53.4% low dose
Ostojic et al. [26]	2016	Serbia	RCT, CO	Guanidinoacetic acid	12	No reported adverse effects	N/A	14	N/A	39.3 (8.8)	N/A	100
Oka et al. [32]	2022	Japan	OPT	6-Methylsulfinylhexyl isothiocyanate	12	No reported adverse effects	N/A	15	N/A	37.5	N/A	80
Teitelbaum et al. [28]	2012	USA	OPT	D-Ribose	3	Nausea, insomnia	N/A	203	N/A	51	N/A	91.6
Teitelbaum et al. [29]	2020	USA	OPT	Porcine serum polypeptide	5	Mild gas and bloating, loose stool, nausea, fatigue, insomnia, feeling irritable and fidgety	N/A	43	N/A	58	N/A	83.7
Venturini et al. [33]	2019	Italy	OPT	Probiotics*	8	Symptom flare-up in one patient, although inflammatory markers did not increase	N/A	9	N/A	NR	N/A	NR

Participant Characteristics

There was a total of 1,046 participants across the 14 studies analyzed in this systematic review who completed their respective clinical interventions. Initially enrolled patients who failed to complete their studies were excluded due to failure to satisfy inclusion criteria, declining to participate, stopping the intervention due to adverse side effects, requesting to leave, or failing to follow up. The proportion of males to females was 19.6% versus 80.4%, respectively. All participants were above 18 years of age, with the average age being 48.4 years.

Interventions on Primary Outcomes

Fatigue was the primary outcome in all studies except two [21,23]. Of the studies that assessed fatigue as the primary outcome, 10 out of 12 reported significant improvements in fatigue levels post-treatment [20,22-31]. While these findings suggest a potential therapeutic benefit, the clinical significance is more difficult to determine due to variability in the fatigue measurement tools used, which included the FIS-40 and FSS. For instance, one study using a combination of CoQ10 and nicotinamide adenine dinucleotide (NADH) demonstrated significantly lower FIS-40 scores [22], and another study using CoQ10 with alpha-lipoic acid also showed decreased fatigue levels on the FSS [20]. Both interventions yielded changes that may be clinically meaningful; however, the lack of standardization in fatigue assessment tools limits the ability to directly compare effect sizes or determine uniform clinical relevance across studies. Treatment with melatonin and zinc supplementation in another study also reported significantly lower FIS-40 scores [23]. CoQ10 plus selenium supplementation was shown to significantly decrease fatigue levels post-intervention [27]. Another study supplementing with hydrogen water found a small but significant difference in perceptive fatigue symptoms using the FSS [24]. A combination treatment of *Ginkgo* and *Cistanche* showed significantly lower levels of perceptive fatigue in both of its treatment groups, with improvement in fatigue being proportional to the treatment dosage [25]. Another open-label trial using anhydrous enol-oxaloacetate (AEO) also decreased patients’ levels of fatigue in its three subgroups, using different dosages and intake frequencies [30]. Treatment with sodium dichloroacetate (DCA) also resulted in significantly lower fatigue levels [31]. Two studies reported no significant difference in fatigue levels after intervention [26,32]. Although treatment with wasabi showed no significant improvements in fatigue levels, it did result in improvements in performance status [32]. In one study, patients receiving D-ribose reported significant improvements in energy, sleep, mental clarity, and overall well-being [28]. Treatment with porcine peptide serum in another study also resulted in significant improvements in energy, well-being, sleep, cognition, anxiety, gastrointestinal health, and pain [29].

One study’s primary endpoint was to assess the efficiency of supplementing with CoQ10 and NADH on maximum heart rate (HR) during a cycle ergometer test [21]. The study reported that participants in the active group had a significant reduction in maximum HR [21]. Another study’s outcome was to assess the safety and efficacy of supplementing with probiotics in ME/CFS patients, reporting significant improvements in mental fatigue levels as well as a decrease in inflammatory cytokines post-treatment [33]. While these findings suggest promise for several interventions, the variability in outcome measures limits the ability to compare effect sizes or determine generalizable clinical efficacy across studies.

Interventions on Secondary Outcomes

Several secondary endpoints were assessed in the studies, including physical function, psychological outcomes, biochemical markers, and QoL domains (Table 2). In the domain of physical function, supplementation with guanidinoacetic acid (GAA) significantly increased muscular creatine levels, muscular strength, and aerobic power, though no significant changes in pain were observed [26]. Treatment with porcine serum polypeptide resulted in a significant reduction in Revised Fibromyalgia Impact Questionnaire (FIQR) scores, indicating improved functional status [29].

**Table 2:** Summary of primary/secondary outcome results *Probiotics: (i) *Enterococcus faecium* and *Saccharomyces boulardii* (Enterelle); *Bifidobacterium longum*, *B. breve*, *B. bifidum*, and *B. infantis* (Bifiselle); (ii) *B. longum* AR81 (Rotanelle); (iii) *Lactobacillus casei* and *B. lactis* (Citogenex); and (iv) *L. rhamnosus* GG and *L. acidophilus* (Ramnoselle). BDI-I & BDI-II: Beck Depression Inventory I & II; CAL: calprotectin; CFQ-11: Chalder Fatigue Scale; DASS-21: Depression Anxiety and Stress Scale; DHEA-S: dehydroepiandrosterone sulfate; FAS: modified fibromyalgia assessment status; FIQR: Revised Fibromyalgia Impact Questionnaire; FIS-40: Fatigue Impact Scale; FSS: Fatigue Severity Scale; HADS: Hospital Anxiety and Depression Scale: HR: heart rate; MFI: Multidimensional Fatigue Inventory; MPQ: McGill Pain Questionnaire; NRS: numerical rating scale; PS: performance status; PSQI: Pittsburgh Sleep Quality Index; PPT: pressure pain threshold; POMS2: Profile of Mood States 2nd edition; SF-36: Short Form Health Survey; SLQQ: Sexual Life Quality Questionnaire; SSS: symptom severity scale; TAC: total antioxidant capacity; UC: urinary free cortisol; TMT-A: Trail-making test; VAS: Visual Analog Scale; WHOQoL: World Health Organization Quality of Life Questionnaire; WPI: widespread pain index; CoQ10: coenzyme Q10; NADH: nicotinamide adenine dinucleotide; HRQoL: health-related quality of life; CRP: C-reactive protein; NS: not significant.

Reference (date)	Treatment intervention	Primary/secondary outcome measures	Results
Barletta et al. (2022) [20]	CoQ10 + alpha-lipoic acid	FSS	Significantly lower (p < 0.0001)
		SSS	Significantly lower (p < 0.0001)
		VAS for pain, sleep, and fatigue	Significantly lower for pain, sleep, and fatigue (p < 0.0001)
		WPI	Significantly lower (p < 0.0001)
		FAS	Significantly lower (p < 0.0001)
Cash and Kaufman (2022) [30]	Anhydrous enol-oxaloacetate (AEO)	CFQ-11	Physical fatigue: significantly lower for 500 mg BID (p < 0.005), 1,000 mg BID (p < 0.005), and 1,000 mg TID (p < 0.01)
Castro-Marrero et al. (2016) [21]	CoQ10 + NADH	Max HR	Significantly lower (p = 0.022)
		FIS-40	Fatigue: significantly lower (p = 0.03)
		MPQ	Pain: NS
		PSQI	Sleep: NS
Castro-Marrero et al. (2021) [22]	CoQ10 + NADH	FIS-40	Fatigue: significantly lower (p < 0.001)
		SF-36	HRQoL: significantly higher (p < 0.05)
			Sleep duration: significantly higher (p = 0.018)
			Habitual sleep efficiency: significantly higher (p = 0.038)
Castro-Marrero et al. (2021) [23]	Melatonin + zinc	FIS-40	Fatigue: significantly lower (p < 0.05)
		SF-36	Sleep: NS
		HADS	Anxiety: NS
		Urinary melatonin metabolite	Significantly higher (p < 0.0001)
Castro-Marrero et al. (2022) [27]	CoQ10 + selenium	FIS-40	Fatigue: significantly lower (p = 0.021)
		SF-36	HRQoL: significantly higher (p = 0.002)
		PSQI	Sleep: NS
		TAC	Significantly higher (p < 0.0001)
		Lipid peroxidase	Significantly lower (p < 0.0001)
		Inflammatory cytokines	NS
Comhaire (2018) [31]	Sodium dichloroacetate	FSS	Fatigue: significantly lower (p = 0.0001)
Friedberg and Choi (2022) [24]	Hydrogen water	FSS	Fatigue: significantly lower (p = 0.04)
		SF-36	Sleep: NS
		DASS-21	NS
Kan et al. (2021) [25]	*Ginkgo* + *Cistanche*	CFQ-11	Fatigue: significantly lower (p = 0.001)
		WHOQoL	HRQoL: significantly higher (p < 0.01)
		SLQQ	Sexual life quality: significantly higher (p < 0.01)
		Blood ammonia	Significantly lower (p < 0.05 low dose; p < 0.01)
		Lactic acid	Significantly lower (p < 0.05 low dose; p < 0.01)
Ostojic et al. (2016) [26]	Guanidinoacetic acid	MFI	Fatigue: NS
		VAS for pain	Pain: NS
		Muscular creatine levels	Significantly higher (p < 0.01)
		Muscular strength and aerobic power	Significantly higher (p < 0.05)
Oka et al. (2022) [32]	6-Methylsulfinylhexyl isothiocyanate	PS	Significantly higher (p = 0.015)
		CFQ-11	Physical and mental fatigue (CFQ-11): NS
		PSQI	Sleep: NS
		PPT	Pain: significantly lower for headache frequency (p = 0.001) & myalgia (p = 0.019)
		TMT-A	TMT-A time: significantly lower (p = 0.007)
			Right occipital PPT: significantly higher (p = 0.01)
		NRS	NRS scores: significantly lower for brain fog (p = 0.011), difficulty finding words (p = 0.015), photophobia (p = 0.008)
		Orthostatic intolerance	Orthostatic intolerance: NS
		POMS2	Significantly higher for vigor (p = 0.045)
		HADS	NS for anxiety & depression
		SF-36	HRQoL: significantly higher for general health perception (p = 0.036) & vitality (p = 0.039)
Teitelbaum et al. (2012) [28]	D-Ribose	7-point hedonic scale for fatigue symptoms	Fatigue symptoms: significantly lower (p < 0.0001)
Teitelbaum et al. (2020) [29]	Porcine serum polypeptide	VAS for fatigue symptoms & pain	Significantly lower for fatigue symptoms (p < 0.001) & pain (p < 0.013)
		FIQR	Significantly lower (p < 0.001)
		Antibody levels	Significantly higher for IgG^a^ (p = 0.008) & IgG1^a ^(p < 0.001)
Venturini et al. (2019) [23]	Probiotics*	UC	2.3x fold increase
		DHEA-S concentration	1.4x fold increase
		CAL	2.5-1x fold increase
		CRP concentration	30% reduction
		SF-36 & CFQ-11	SF-36 & CFQ-11 combination: significant improvement of mental component (p = 0.043)
		BDI-I & BDI-II	NS

Regarding psychological and clinical outcomes, CoQ10 and alpha-lipoic acid supplementation improved sleep and pain, as well as scores on the widespread pain index (WPI) and the modified fibromyalgia assessment status [20]. Of the two studies using CoQ10 and NADH, one reported improved fatigue with no change in pain or sleep, while the other reported significant improvement in sleep and health-related QoL (HRQoL) [21,22]. Melatonin and zinc supplementation led to increased melatonin metabolite excretion but did not improve sleep or anxiety, and hydrogen water treatment showed no significant changes in secondary outcomes [23,24]. Wasabi (6-methylsulfinylhexyl isothiocyanate (6-MSITC)) improved self-reported vigor and reduced symptoms such as brain fog, photophobia, and word-finding difficulty [32].

Biochemical outcomes were assessed in several trials. CoQ10 and selenium supplementation increased total antioxidant capacity (TAC) and reduced lipid peroxidase levels, although it had no impact on inflammatory cytokines [27]. Probiotic supplementation resulted in biomarker changes including increased urinary free cortisol (UC), fecal calprotectin (CAL), and dehydroepiandrosterone sulfate (DHEA-S) and decreased C-reactive protein (CRP) [33].

In terms of QoL, *Ginkgo* and *Cistanche* significantly improved both general health-related and sexual QoL, along with reductions in blood ammonia and lactic acid levels [25]. Wasabi supplementation was also associated with improved vitality-related HRQoL, and CoQ10 with selenium increased HRQoL even in the absence of changes to sleep quality [27,32]. The D-ribose study did not report any secondary endpoints [28].

Quality Assessment

Quality assessment scores for each study can be found in Supplemental materials 1 and 2. Six of the studies in this review were determined to be of high quality based on the Delphi list (Delphi score > 7) [21-26] while only one study assessed using the QuADS criteria met the threshold for good quality (QuADS score = 32) [27]. Lower-quality studies commonly failed to implement randomization and blinding of participants and assessors, which are key methodological steps that help reduce selection and measurement bias. Item nine of the Delphi list, which assesses the intention-to-treat (ITT) statement, was the least addressed item among the RTCs included in this review. The absence of ITT analysis may overestimate treatment effects by excluding non-compliant participants. For the OPTs, items six and 10 from the QuADS criteria were the least addressed. These items assessed the rationale behind the data collection tools used and the justification for the analytic method selected, respectively. The absence of clear theoretical justification for these aspects reduces both the interpretability and reproducibility of study findings. These methodological gaps are important to consider when evaluating the reliability and validity of reported outcomes. Trials that lack blinding are more prone to expectation biases, and those without robust analytical rationale may yield spurious or non-generalizable associations. Future research in this area would benefit from the routine use of ITT protocols and the adoption of validated, theory-driven outcome measures to enhance methodological rigor and cross-study comparability.

Discussion

ME/CFS is a debilitating medical condition with no established standard of care [5]. Consequently, many patients resort to either clinician- or self-recommended dietary and nutritional supplements to alleviate symptoms [1,5]. This retrospective systematic review reviewed data from 14 trials reporting improvements in self-reported or administered measures of disease severity and other symptoms of illness.

The heterogeneity of the instruments used in these studies presents a challenge, however, to conduct a comparison of the effectiveness of treatments. The U.S. NIH’s National Institute of Neurological Disorders and Stroke (NINDS) collaborated with the CDC in 2018 to identify and define common data elements (CDEs) for ME/CFS to reduce this prevalent issue in translational research [34,35]. However, none of the studies included in this review employed the recommended CDEs, which limits comparability across trials and contributes to inconsistency in reported outcomes. This methodological shortcoming compromises the ability to synthesize evidence and draw generalizable conclusions. In addition, clinical trial registries, such as the National Library of Medicine ClinicalTrials.gov registry, provide valuable information on the effectiveness of the treatments while avoiding information biases often associated with systematic reviews. Although the requirements for registering studies are designed to standardize the information within a study record including the type of study, intervention, trial phase, funding source, outcomes, and data types to be reported, the Food and Drug Administration Amendments Act (FDAAA 801) mandates the registration of only studies that meet the definition of an "applicable clinical trial" (ACT) onto ClinicalTrials.gov [36,37]. None of the studies included in this review reported their results on ClinicalTrials.gov or have utilized the core instruments recommended by the CDEs. The absence of trial registration introduces risks of publication bias, selective outcome reporting, and overestimated effect sizes, which can weaken the transparency and reproducibility of findings. This study retrieved applicable results from separate journals and databases, including PubMed, Cochrane, Medline, and Web of Science, to complete this review. Not only is this process of scientific evaluation cumbersome, but it is also impractical for a patient population with a significant disease burden to make an informed decision.

This review highlights the already reported sex differences in ME/CFS affliction, with 80.4% of the aggregated study population being female, potentially attributed to underlying neuroendocrine adaptations. However, only three studies reported race or ethnicity, and all participants in those studies identified as Caucasian [22,23,27]. This lack of diversity restricts the generalizability of findings and overlooks possible race or ethnicity-specific manifestations of ME/CFS. Moreover, many of the studies included in this review were conducted outside of the United States and originated from a single institution, underscoring the need for more diverse studies to confirm replicability and ensure population representation. Studies across multiple therapeutic areas have recognized this general lack of racial/ethnic diversity in research studies, potentially due to identified fiscal and sociopsychological barriers faced by underserved communities. The lack of representation hinders the profiling of the crucial phenotypic traits of illnesses that may be race/ethnicity-specific, leading to worse public health outcomes in these communities.

All participants in this review were diagnosed with ME/CFS using the CDC diagnostic criteria from 1994 and the 2015 National Academy of Medicine criteria [38]. More specific diagnostic criteria such as the Canadian Consensus Criteria (CCC) and International Consensus Criteria (ICC) should be considered in future research to minimize diagnostic overlap with other conditions [2,39]. Furthermore, several studies failed to implement key methodological practices such as blinding of participants and assessors or the use of ITT analysis. These flaws may introduce bias, reduce internal validity, and compromise the reliability of treatment effects observed in unblinded or non-randomized trials.

Despite these limitations, the studies discussed represent the core body of available evidence on this topic across databases such as ClinicalTrials.gov, PubMed, Medline, Cochrane, and Web of Science. The focus of this review was studies utilizing supplements/nutrients/diet as the sole intervention, although it is worth noting that one trial explored the effects of a mitochondria-support nutrient formula coadministered with a low-dose stimulant [40,41]. This combination had synergistic effects, optimizing cellular energy production and alertness while improving tolerability and reducing reliance on stimulants. However, this was not representative of the broader evidence base reviewed and falls outside the review’s inclusion criteria. Therefore, recommendations regarding combination therapy with supplements and medications remain speculative and should be interpreted with caution, requiring the need for future studies.

Finally, further investigations employing the CDEs for ME/CFS are needed not only to standardize measured outcomes but also to enhance reproducibility, reduce bias, and enable direct comparison across trials. Incorporating CDEs, such as uniform fatigue scales, cognitive testing protocols, and biological markers, could provide more structured and interpretable data. High-quality, rigorously designed RCTs with transparent reporting and diverse populations are essential to strengthen the evidence on the role of nutraceuticals in ME/CFS and guide future clinical recommendations.

## Conclusions

This review identified current studies showing that supplementation with CoQ10, NADH, selenium, melatonin, AEO, alpha-lipoic acid, DCA, and zinc may be beneficial in improving subjective fatigue symptoms in patients with ME/CFS. Additionally, supplements such as *Ginkgo*, *Cistanche*, wasabi, and probiotics have the potential to alleviate symptoms related to cognitive and memory impairment, while GAA may positively affect muscular strength. CoQ10, selenium, and probiotics have also demonstrated anti-inflammatory effects. While these findings are encouraging, they must be interpreted with caution, given the considerable methodological limitations present in many of the included studies. The frequent absence of blinding, lack of ITT analysis, limited racial/ethnic representation, and heterogeneity in outcome measures significantly compromise internal validity and generalizability.

## Appendices

Supplemental material 1

**Table 3:** Delphi list quality assessment of RCTs ^a^The trial was randomized ^b^Treatment allocation was concealed ^c^Groups were similar at baseline regarding the most important prognostic indicators ^d^Eligibility criteria were specified ^e^Outcome assessor was blinded ^f^Care provider was blinded ^g^Patients were blinded ^h^Point estimates and measures of variability were presented for the primary outcome measures ^i^Analysis included an intention-to-treat analysis RCTs: randomized controlled trials.

References	Randomized^a^	Treatment allocation concealed^b^	Groups similar at baseline^c^	Eligibility criteria specified^d^	Outcome assessor blinded^e^	Care provider blinded^f^	Patient blinded^g^	Variability measures^h^	Intention-to-treat analysis^i^	Total score (0-9)
Barletta et al. (2022) [20]	1	0	1	1	0	0	1	1	0	6
Castro-Marrero et al. (2016) [21]	1	1	1	1	0	1	1	1	1	8
Castro-Marrero et al. (2021) [22]	1	1	1	1	0	1	1	1	1	8
Castro-Marrero et al. (2021) [23]	1	1	1	1	0	1	1	1	0	7
Friedberg & Choi (2022) [24]	1	1	1	1	0	1	1	1	0	7
Kan et al. (2021) [25]	1	1	1	1	0	1	1	1	0	7
Ostojic et al. (2016) [26]	1	1	0	1	0	1	1	1	1	7

Barletta et al., 2022 [20]

a. The trial was randomized

b. Treatment allocation was not concealed

c. Groups were similar at baseline

d. Inclusion criteria were specified

e. Outcome assessor was not blinded

f. Care provider was not blinded

g. Patient was blinded

h. Point measures and measures of variability are provided

i. Analysis did not include an intention-to-treat analysis

Castro-Marrero et al., 2016 [21]

a. The trial was randomized

b. Patients were randomized in a double-blind fashion, 1:1 ratio (computer-generated list using STATA 9.0)

c. Groups were similar at baseline

d. Eligibility criteria were specified. Inclusion criteria included ME/CFS patients who met the 1994 CDC case criteria. Exclusion criteria were contraindication of an ergometer exercise test, participation in other trials in the 30 days prior to inclusion, intake of any drug or banned substances (statins, dietary supplements, anti-hypertension, or beta-blocker drugs), pregnancy or breast-feeding, secondary hypertension, hepatobiliary tract disease, cardiovascular or pulmonary disorder, and inability to communicate and comply with all study requirements

e. N/A

f. Yes, it was a double-blind study

g. Yes, it was a double-blind study

h. Point measures and measures of variability are provided

i. Yes, statistical analysis included an intention-to-treat analysis

Castro-Marrero et al., 2021 [22]

a. The trial was randomized.

b. Patients were randomized in a double-blind fashion, 1:1 ratio (independent investigator not involved in the intervention using the result of a list of random numbers generated by a computer program)

c. Groups were similar at baseline

d. Eligibility criteria were specified. Inclusion criteria included females, aged 18 or older, and ME/CFS patients who met the 1994 CDC case criteria. Exclusion criteria were any active medical condition that explained the chronic fatigue, previous diagnosis not unequivocally resolved, past or current neuropsychiatric disorders, participation in another clinical trial within 30 days prior to study inclusion, inability to follow instructions, failure to provide signed informed consent, consumption of certain drugs/supplements that might influence outcome measures in the last 90 days or whose withdrawal might be a relevant problem, anticoagulant treatment, pregnancy or breast-feeding, smoking, alcohol intake or substance abuse, obesity (BMI > 30 kg/m^2^), and hypersensitivity to CoQ10 or NADH

e. N/A

f. Yes, it was a double-blind study

g. Yes, it was a double-blind study

h. Point measures and measures of variability are provided

i. Yes, statistical analysis included an intention-to-treat analysis

Castro-Marrero et al., 2021 [23]

a. The trial was randomized

b. Patients were randomized in a double-blind fashion, 1:1 ratio (independent investigator not involved in the intervention using a table of random numbers in the Milton statistical guide)

c. Groups were similar at baseline

d. Eligibility criteria were specified. Inclusion criteria included females, aged 18-65, and ME/CFS patients who met the 1994 CDC/Fukuda case criteria and provided signed informed consent. Exclusion criteria were any active medical condition that explained the chronic fatigue, previous diagnosis not unequivocally resolved, past or current neuropsychiatric disorders, participation in another clinical trial within 30 days prior to study inclusion, inability to follow instructions, failure to provide signed informed consent, current consumption of medications that may interfere with the results and/or whose withdrawal may be a relevant problem, anticoagulant treatment, pregnancy or breast-feeding, use of oral contraceptives or other hormonal preparations in the previous six months, smoking, alcohol intake or substance abuse, and severe obesity

e. N/A

f. Yes, it was a double-blind study

g. Yes, it was a double-blind study

h. Point measures and measures of variability are provided

i. No, statistical analysis did not include an intention-to-treat analysis

Friedberg and Choi, 2022 [24]

a. The trial was randomized

b. Patients were randomized in a double-blind fashion, 1:1 ratio (computer-generated randomization)

c. Groups were similar at baseline with respect to sex or illness duration, but the active group was younger than the placebo group

d. Eligibility criteria were specified. Inclusion criteria included males and females aged 18-65, not pregnant, physically capable and willing to perform the study tasks, with at least six months of persistent and unremitting fatigue, presenting symptom and impairment criteria for ME/CFS according to the 1994 CDC/Fukuda guidelines, and experiencing at least four out of eight secondary symptoms

e. N/A

f. Yes, it was a double-blind study

g. Yes, it was a double-blind study

h. Point measures and measures of variability are provided

i. No, statistical analysis included an intention-to-treat analysis

Kan et al., 2021 [25]

a. The trial was randomized

b. Patients were randomized in a double-blind fashion-sequence generator randomization

c. Groups were similar at baseline

d. Eligibility criteria were specified. Inclusion criteria included males and females aged 35-60 and ME/CFS patients who met the 1994 CDC/Fukuda case criteria. Exclusion criteria included BMI ≥ 28 kg/m^2^, flulike/viral infection symptoms within three months prior to the first visit to the clinical site, a history/diagnosis of any condition that could affect study results, current use of medicine for cardiovascular or metabolic diseases, smoking, a history of alcohol abuse, pregnancy and lactation, use of nutritional therapies that promoted exercise capacity within three months before screening, weight loss/gain of over 5 kg within three months before screening, hospitalization within three months of screening, participation in similar clinical trials within six months before screening, and willingness to comply with study procedures

e. N/A

f. Yes, it was a double-blind study

g. Yes, it was a double-blind study

h. Point measures and measures of variability are provided

i. No, statistical analysis included an intention-to-treat analysis

Ostojic et al., 2016 [26]

a. The trial was randomized

b. Randomization was computer-generated

c. Differences between groups at baseline are not provided

d. Inclusion criteria include participants who were 18 and over and fulfilled the 1994 Fukuda definition. Exclusion criteria included psychiatric comorbidity, use of any dietary supplement within four weeks of the study period, unwillingness to attend to follow-up analysis, and pregnancy

e. N/A

f. Yes, it was a double-blind study

g. Yes, it was a double-blind study

h. Point measures and measures of variability are provided

i. Yes, statistical analysis included an intention-to-treat analysis

Supplemental material 2

**Table 4:** QuADS quality assessment for OPTs ^a^Theoretical or conceptual underpinning to the research ^b^Statement of research aim ^c^Clear description of research setting and target population ^d^Eligibility criteria were specified ^e^Outcome assessor was blinded ^f^Care provider was blinded ^g^Patients were blinded ^h^Point estimates and measures of variability were presented for the primary outcome measures ^i^Analysis included an intention-to-treat analysis ^j^Analytic method selected was justified ^k^The method of analysis was appropriate to answer the research aim/s ^l^Evidence that the research stakeholders were considered in the research design or conduct ^m^Strengths and limitations were critically discussed OPTs: open-labelled pilot trials.

References	Underpinning^a^	Research aim^b^	Setting & target population^c^	Appropriate design^d^	Appropriate sampling^e^	Rationale for data collection tools^f^	Format & content of data collection tool^g^	Data collection procedure^h^	Recruitment data^i^	Analytic method justification^j^	Analytic method appropriate^k^	Stakeholders considered in research design^l^	Discussion of strengths & limitations^m^	Total score (0-39)
Cash and Kaufman (2022) [30]	3	3	2	2	1	1	2	2	3	1	1	0	0	21
Castro-Marrero et al. (2022) [27]	3	3	3	2	3	1	3	3	3	1	3	1	3	32
Comhaire (2018) [31]	3	2	2	1	2	1	2	1	1	1	1	0	2	19
Oka et al. (2022) [32]	3	2	3	3	1	2	3	1	3	3	3	1	3	31
Teitelbaum et al. (2012) [28]	3	3	2	1	2	1	1	2	1	0	3	0	3	22
Teitelbaum et al. (2020) [29]	3	3	2	1	2	1	2	2	1	1	3	1	1	23
Venturini et al. (2019) [33]	3	2	3	2	2	1	2	2	3	0	3	0	0	23

Cash and Kaufman, 2022 [30]

a. Explicit discussion of the theories or concepts that inform the study, with application of the theory or concept evident through the design, materials, and outcomes explored

b. Explicit and detailed statement of aim/s in the main body of the report

c. Description of research setting is made but is lacking detail

d. The study design can address the stated research aim, but a double-blind randomized clinical trial would have been a more suitable alternative

e. Evidence of consideration of the sample required

f. Very limited explanation for the choice of data collection tool

g. Structure and/or content of tool/s allow for data to be gathered broadly addressing the stated aim/s but could benefit from refinement

h. Study stated stages of data collection procedure but with limited detail

i. Complete data allowing for a full picture of recruitment outcomes were provided

j. Very limited justification for the choice of the analytic method selected

k. Method of analysis can only address the research aim/s basically or broadly

l. No mention at all

m. No mention at all

Castro-Marrero et al., 2022 [27]

a. Explicit discussion of the theories or concepts that inform the study is given, with application of the theory or concept being evident through the design, materials, and outcomes explored

b. Explicit and detailed statement of aim is provided in the main body of the report

c. Specific description of the research setting and target population of the study was provided

d. The study design can address the stated research aim, but a double-blind randomized clinical trial would have been a more suitable alternative

e. Detailed evidence of consideration of the sample required to address the research aim was provided

f. Very limited explanation for choice of data collection tools was provided

g. Structure and content of tool/s allow for detailed data to be gathered around all relevant issues required to address the stated research aim

h. Detailed description of each stage of the data collection procedure including when, where, and how data were gathered such that the procedure could be replicated was given

i. Complete data allowing for the full picture of recruitment outcomes were provided

j. Very limited justification for the choice of the analytic method selected was provided.

k. The method of analysis selected is the most suitable approach to attempt to answer the research aim/s in detail

l. Evidence that the research stakeholders were considered in research design or conduct was weak

m. Strengths and limitations were critically and thoroughly discussed

Comhaire, 2018 [31]

a. Explicit discussion of the theories or concepts that inform the study, with application of the theory or concept evident through the design, materials, and outcomes explored

b. Aims statement made but may only appear in the abstract or be lacking detail

c. Description of research setting is made but is lacking detail

d. The study design can only address some aspects of the stated research aim

e. Evidence of consideration of the sample required to address the research aim was provided

f. Very limited explanation for the choice of data collection tool

g. Structure and/or content of tool/s suitable to address some aspects of the research aim/s or to address the aim/s superficially

h. Basic and brief outline of data collection procedure

i. Minimal and basic recruitment data

j. Very limited justification for the choice of the analytic method selected

k. Method of analysis can only address the research aim/s basically or broadly

l. No mention at all

m. Discussion of some of the key strengths and weaknesses of the study but not in depth and with consideration for future trials

Oka et al., 2022 [32]

a. Explicit discussion of the theories or concepts that inform the study, with application of the theory or concept evident through the design, materials, and outcomes explored

b. Aims statement made but may only appear in the abstract or be lacking detail

c. Specific description of the research setting and target population of the study

d. The study design selected appears to be the most suitable approach to attempt to answer the stated research aim/s

e. Evidence of consideration of the sample required

f. Basic explanation of rationale for choice of data collection tool/s

g. Structure and content of tool/s allow for detailed data to be gathered around all relevant issues required to address the stated research aim/s

h. Basic and brief outline of data collection procedure

i. Complete data allowing for a full picture of recruitment outcomes

j. Detailed justification for the choice of the analytic method selected

k. Method of analysis selected is the most suitable approach to attempt to answer the research aim/s in detail

l. Consideration of some of the research stakeholders

m. Thorough discussion of strengths and limitations of all aspects of the study including design, methods, data collection tools, sample, and analytic

Teitelbaum et al., 2012 [28]

a. Explicit discussion of the theories or concepts that inform the study is given, with application of the theory or concept being evident through the design, materials, and outcomes explored

b. Explicit and detailed statement of the aim is provided in the main body of the report

c. Description of the research setting was made, but it was lacking detail

d. The study design can only address some aspects of the stated research aim

e. Evidence of consideration of the sample required to address the research aim was provided

f. Very limited explanation for the choice of data collection tools was provided

g. Structure and content of collection tool/s were suitable to address some aspects of the research aim or to address the aim superficially

h. Study stated stages of the data collection procedure but with limited detail

i. Minimal and basic recruitment data were provided

j. No justification for the analytic method selected was provided

k. The method of analysis selected is the most suitable approach to attempt to answer the research aim/s in detail

l. No mention that the research stakeholders were considered in the research design or conduct

m. Strengths and limitations were critically and thoroughly discussed

Teitelbaum et al., 2020 [29]

a. Explicit discussion of the theories or concepts that inform the study is given, with application of the theory or concept being evident through the design, materials, and outcomes explored

b. Explicit and detailed statement of aim is provided in the main body of the report

c. Description of the research setting was made, but it was lacking detail

d. The study design can only address some aspects of the stated research aim

e. Evidence of consideration of the sample required to address the research aim was provided

f. Very limited explanation for the choice of data collection tools was provided

g. Structure and content of collection tool/s allowed for data to be gathered broadly addressing the stated aim but could benefit from refinement

h. Study stated stages of data collection procedure but with limited detail

i. Minimal and basic recruitment data were provided

j. Very limited justification for the analytic method selected was provided

k. The method of analysis selected is the most suitable approach to attempt to answer the research aim/s in detail

l. Evidence that the research stakeholders were considered in research design or conduct was weak

m. Strengths and limitations were minimally discussed, with omissions of many key issues

Venturini et al., 2019 [33]

a. Explicit discussion of the theories or concepts that inform the study is given, with application of the theory or concept being evident through the design, materials, and outcomes explored

b. Aims statement made but may only appear in the abstract or be lacking detail

c. Specific description of the research setting and target population of study, e.g., nurses and doctors from GP practices in [x] part of [x] city in [x] country

d. The study design can address the stated research aim/s, but there is a more suitable alternative that could have been used in addition, such as longer administration of medication and a control group to compare the intervention to

e. Evidence of consideration of the sample required to address the research aim was provided

f. A very limited explanation for the choice of data collection tools was provided

g. Structure and/or content of tool/s allow for data to be gathered broadly addressing the stated aim/s but could benefit from refinement given the broad range of values that could address the rather broad claim

h. States each stage of the data collection procedure but with limited detail or states some stages in detail but omits others

i. Complete data allowing for a full picture of recruitment outcomes were provided

j. No mention of the rationale for the analytic method chosen

k. The method of analysis selected is the most suitable approach to attempt to answer the research aim/s in detail

l. No mention that the research stakeholders were considered in the research design or conduct

m. No critical evaluation of the strengths and weaknesses of the study at all
